# The impact of cardiometabolic multimorbidity on cognitive function in older adults: a review

**DOI:** 10.3389/fpubh.2026.1795161

**Published:** 2026-03-23

**Authors:** Huan Liu, Junxiang Sun, Ting Wang

**Affiliations:** Nursing Department, Sir Run Run Shaw Hospital, Zhejiang University School of Medicine, Hangzhou, China

**Keywords:** cardiometabolic multimorbidity, cognitive function, older adults, impact, review

## Abstract

Cardiometabolic multimorbidity (CMM) represents the most prevalent and most detrimental chronic disease comorbidity pattern for cognitive impairment, directly accelerating cognitive dysfunction and dementia progression. However, existing research predominantly investigates cognitive function in patients with single diseases or specific binary comorbidities, leaving significant gaps in understanding the cognitive domain among older adults with CMM. This review focuses on the impact of CMM on cognitive function in the older adults synthesizing perspectives on their correlation, underlying mechanisms, assessment tools, and risk factors. It aims to provide insights for screening high-risk populations and formulating management strategies to delay the risk of cognitive dysfunction.

## 1 Introduction

Multimorbidity refers to the coexistence of two or more chronic diseases in the same individual. Cardiometabolic multimorbidity (CMM) refers to the coexistence of two or more cardiometabolic diseases (CMDs), including heart disease, diabetes, stroke, etc. (1). However, the spectrum of diseases included varies across studies. While most studies have focused on ischemic heart disease, stroke, and diabetes mellitus (1, 2), some have additionally incorporated hypertension or simultaneously included dozens of cardiometabolic diseases. Consequently, a standardized definition for CMM is currently lacking (3). CMM is becoming increasingly prevalent with population aging. Studies indicate that the prevalence of chronic disease comorbidity among Chinese seniors is 43.65%, while the prevalence of CMM in this population reaches 19.67%, making it the most common chronic disease comorbidity pattern (4). Individual CMDs have been established as independent risk factors for cognitive impairment. Recently, a growing body of evidence suggests a potential dose–response relationship between the combined effect of CMM and cognitive impairment (5, 6). A large-scale 18-year Swedish cohort study found that among various chronic disease comorbidity patterns, patients with CMM exhibited the most significant increased risk of progressing from normal cognitive function to cognitive impairment, dementia, and ultimately death (HR 1.70, 95% CI 1.15–2.52) (7). CMM dramatically accelerates cognitive impairment and dementia progression (8), thereby increasing complication rates, hospitalization-related financial toxicity, reducing quality of life, and directly elevating mortality in older patients (9). The risk of cognitive impairment is heightened in older adults with CMM, and the presence of cognitive impairment in these patients leads to diminished self-management capabilities, creating a vicious cycle (10). Previous research has largely concentrated on the relationship between a single or specific pair of CMDs and cognitive function, overlooking the cumulative effects of different CMM combinations and quantities on cognitive function. The relationship between CMM and cognitive function is gaining increasing attention from scholars, emerging as a critical research focus both domestically and internationally. This review examines the correlation between CMM and cognitive function, its underlying mechanisms, assessment tools, and risk factors, providing a theoretical foundation for early prevention and intervention of cognitive impairment in older adults with CMM.

## 2 Literature retrieval strategy

A systematic literature search was performed in both Chinese and English databases using the following keywords: cardiometabolic, cardiometabolic multimorbidity, CMM, multimorbidity, multiple chronic conditions, concurrent chronic conditions, concurrent chronic disorders, combined with cognitive, cognition, dementia. Chinese databases included Wanfang, CNKI, CBM, and VIP. English databases included Cochrane Library, PubMed, CINAHL, Web of Science, and Ovid.

The search was conducted from database inception to October 2025. Studies were included if they investigated the association between CMM and cognitive impairment in older adults and were published in Chinese or English. And mainly focuses on observational studies. Studies were excluded if they were irrelevant to the topic, of low methodological quality, or unavailable in full text.

## 3 Correlation between CMM and cognitive function in older patients

Numerous large-scale cohort studies have demonstrated that CMM is significantly and positively associated with an increased risk of cognitive impairment in older patients, directly accelerating dementia progression, indicating a dose-dependent relationship (5, 6, 11, 12). The number of comorbid CMDs exhibits an additive effect on cognitive impairment, meaning that the risk of cognitive impairment increases with a greater number of CMDs, a finding that has been confirmed in patients with multimorbidity (13). Compared to older adults without CMDs, the risk of cognitive impairment shows a clear upward trend in those with 1, 2, and 3 or more CMDs (4, 14). Moreover, older adults with 3 or more CMDs exhibit the most significant decline in scores across all cognitive domains (5). However, there are certain limitations in exploring the relationship between the number of CMDs and cognitive dysfunction. For instance, the methods used to quantify the burden of comorbidities in the aforementioned literature vary significantly. Common measures of comorbidity burden, such as the Charlson Comorbidity Index or simple disease count, are limited in their effectiveness because they cannot establish a direct connection between specific diseases and outcomes. In addition, as noted in studies like Dove et al., reliance on healthcare databases introduces the potential for surveillance bias; individuals with CMM have more frequent clinical encounters, which may artifactually inflate the incidence of diagnosed cognitive impairment. Furthermore, different CMM comorbidity patterns are also associated with cognitive impairment, although current research on this topic, both domestically and internationally, is limited and has yet to yield consistent conclusions. In China, the most common dyadic CMM patterns are heart disease comorbid with hypertension, followed by hypertension comorbid with diabetes, hypertension comorbid with cerebrovascular disease, and heart disease comorbid with diabetes. Among these, comorbidity patterns involving hypertension are associated with a higher risk of cognitive impairment in older adults (4). A large 14-year cohort study in Europe reported that the most common CMM patterns were myocardial infarction comorbid with diabetes, cerebrovascular disease comorbid with diabetes, cerebrovascular disease comorbid with myocardial infarction, and diabetes comorbid with both cerebrovascular disease and myocardial infarction. Another large European multi-cohort study spanning 14 countries found that among binary CMM patterns, cerebrovascular disease comorbid with diabetes was associated with the most significant decline in cognitive function scores in older adults (5). Additionally, a 12-year large-scale cohort study by Dove et al. (8) found that among CMM patients, cognitive impairment had a more detrimental impact in early old age (60–78 years) compared to late old age (≥78 years) (8). The study by Dove et al. benefits from a unique, genetically informative twin study design, which facilitates the exploration of the role of CMM in its association with cognitive function within a genetic context. However, the database only includes inpatient and outpatient care records, lacking access to primary care data. Furthermore, individuals with CMM may seek medical services more frequently than those without, thereby increasing their likelihood of being diagnosed with cognitive impairment. This could potentially lead to an overestimation of the study findings. However, most of these studies have been conducted in localized areas within high-income countries (e.g., North America and Europe) and have predominantly focused on geriatric populations, while the assessment methods for multimorbidity also vary across studies. Therefore, it is necessary to employ scientifically sound clustering approaches to investigate the relationship between CMM patterns and cognitive impairment across countries with varying income levels and among different demographic groups (e.g., age and gender). This will provide an evidence-based population screening criterion and data support for future targeted intervention practices. Understanding the association between different CMM comorbidity patterns and the risk of cognitive impairment is crucial. It helps elucidate the pathophysiological mechanisms through which different diseases influence cognitive function and provides scientific basis for developing precision management strategies tailored to the comorbidity characteristics of CMM patients.

## 4 Mechanisms underlying cognitive impairment in older patients with CMM

While well-established research exists on the mechanisms by which individual diseases such as cardiovascular disease, cerebrovascular disease, and diabetes cause cognitive impairment (15–17), studies on the pathophysiological mechanisms linking CMM to cognitive decline remain in their infancy. The underlying mechanisms remain unclear and require further experimental investigation. Recent evidence suggests that cognitive impairment may be associated with atherosclerotic processes; although the underlying mechanisms are not fully elucidated, proposed pathways include increased arterial pulsatility-induced cerebral microvascular damage, reduced cerebral blood flow and oxygenation leading to dementia-related atrophy, hippocampal neurodegeneration associated with carotid wall thickening, and cerebral hypoperfusion secondary to silent embolization. Ultimately, it results in impaired synaptic function, neuronal apoptosis, and brain structural remodeling, which collectively mediate the decline of cognitive domains such as memory and executive function (18). The prevailing view suggests that inflammation-related neurodegenerative processes and early non-specific biomarkers of brain injury may be involved. A recent large-sample cross-sectional study from a hospital in Shandong Province, focusing on older rural Chinese adults with CMM, reported that plasma amyloid-beta (Aβ) and neurofilament light chain (NfL) mediate cognitive impairment in this population by regulating neurodegenerative pathways (19). Plasma Aβ and NfL are established non-specific biomarkers for neurodegeneration and neuroaxonal injury (20, 21). The association between dementia risk in CMM patients and increased plasma Aβ and NfL may be attributed to CMM-related vascular inflammation leading to endothelial dysfunction and increased blood–brain barrier permeability. This allows more plasma Aβ and NfL to cross into the brain, potentially contributing to neurodegenerative changes. Another large cohort study funded by the National Natural Science Foundation of China supports the role of protein-related biomarkers in mediating neuronal damage. It found that increased levels of phosphorylated tau protein markers in cerebrospinal fluid are significantly positively correlated with cognitive impairment in CMM patients, ultimately progressing to dementia (6). Some perspectives suggest that coexisting multiple diseases with high cognitive impairment risk may produce synergistic effects, exacerbating cognitive decline (22). This leads to increased risks of cognitive impairment and dementia in older CMM patients as the number of comorbidities rises. Furthermore, compared to patients with a single disease, older CMM patients face polypharmacy due to multimorbidity and advanced age. Studies indicate that polypharmacy itself can increase the risk of cognitive dysfunction (23).

Increasing evidence indicates that CMM exerts divergent effects on different subtypes of dementia (19, 24). Compared with Alzheimer’s disease, CMM demonstrates a much stronger association with vascular dementia, mainly through cerebral small vessel disease, chronic hypoperfusion, white matter disruption, and ischemic injury. Moreover, the coexistence of vascular risk factors and neurodegenerative pathology significantly increases the risk of mixed dementia, suggesting a synergistic interaction between cardiometabolic disorders and underlying brain damage. These observations highlight that CMM contributes differentially to distinct dementia subtypes and underscore the importance of early and integrated management of vascular and metabolic risks for preventing both vascular and mixed dementia in later life. Research has indicated that CMDs are associated with domain-specific cognitive impairment, with significant heterogeneity across distinct cognitive domains. Executive function and processing speed are the earliest and most severely affected cognitive domains, while delayed recall and visuospatial ability also decline to varying degrees, especially in individuals with comorbidities such as stroke (25). However, research on the impact of CMM on cognitive function remains in the preliminary stage. Existing studies have mainly focused on the effects of different types of CMM, age, sex, and the number of comorbidities on cognitive function, and it has been found that there is a decline in the overall cognitive domain scores. However, few studies have systematically explored its domain-specific effects on various cognitive domains, which warrants further in-depth investigation in the future.

In summary, inflammation serves as a convergence point for the aforementioned mechanisms and plays a pivotal role in the pathogenesis of CMM. However, research into the underlying mechanisms of cognitive impairment in patients with CMM remains in its preliminary stages both domestically and internationally. The potential mechanisms underlying the association between CMM and cognitive phenotypes remain unclear. The precise biological pathways through which CMM patterns influence cognitive impairment also require further clarification. Existing literature remains limited, necessitating additional experimental studies to elucidate these mechanisms.

## 5 Cognitive assessment tools for older patients with CMM

Currently, there are no cognitive assessment tools specifically developed for older adults with CMM. Existing studies predominantly employ screening tools intended for single-disease cognitive assessment. Guidelines recommend neuropsychological testing as the most commonly used assessment tool, which evaluates overall cognitive function, individual cognitive domains, activities of daily living, and mental/psychological status (26). This is supplemented by imaging studies and laboratory tests for further evaluation and differential diagnosis, a methodology already widely applied in single cardiovascular diseases. The Mini-Mental State Examination (MMSE), valued for its simplicity and brevity, is particularly suitable for geriatric populations and has become the most frequently used screening tool in cognitive function studies older adult patients with CMM both domestically and internationally (4, 6, 27, 28). Other commonly used assessment tools include the Montreal Cognitive Assessment (MoCA) and the Basic Cognitive Ability Test (BCAT) (29). Additionally, international studies favor tests such as the Auditory Verbal Learning Test and the Clock Drawing Test to evaluate patients’ memory, language, and spatial cognition (5, 6). However, existing studies predominantly assess cognitive function through either overall cognitive performance, single cognitive domains, activities of daily living, or mental health status, which failing to meet guideline recommendations and potentially leading to over- or underestimation of outcomes. A 2020 Lancet article first proposed considering the association between different CMM combinations and cognitive impairment, emphasizing the need for personalized, multidimensional assessment of cognitive impairment in CMM patients (30). Older CMM patients may experience multidimensional cognitive impairment due to the cumulative effects of the disease, suggesting a greater need for multi-dimensional cognitive screening in this population. Developing a cognitive assessment tool suitable for patients with multimorbidity, particularly CMM, is imperative.

## 6 Risk factors for cognitive impairment in older patients with CMM

### 6.1 Sociodemographic factors

Studies indicate that being male and receiving a CMM diagnosis in middle age are risk factors for cognitive impairment (5). A cross-sectional survey of 5,765 older adult patients with CMM in rural China revealed a stronger association between CMM diagnosis and cognitive impairment in males than females (19). This may stem from stronger correlations between CMM comorbidity patterns, metabolic disorders, and total brain tissue/gray matter volume in males compared to females. Research indicates that the CMM pattern, compared to other chronic disease co-morbidity patterns, is significantly associated with smaller brain tissue (31). Given that men exhibit higher CMM prevalence than women, and that female CMM patients may pursue healthier lifestyles and seek healthcare more actively, reducing the impact of co-morbidities on brain atrophy in women compared to men, male CMM patients have smaller total brain tissue and gray matter volumes than females. This may explain the observed gender difference. An 18-year Swedish twin cohort study revealed that individuals diagnosed with CMM in middle age face a higher risk of cognitive impairment than younger or older adults (7). This may also relate to the stronger association between CMM and brain volume observed in middle-aged individuals compared to younger or older age groups. These findings were further validated in patients with chronic disease co-morbidity (13). Therefore, implementing targeted interventions in middle-aged individuals with comorbidities is crucial for preventing or delaying cognitive impairment. Increased attention should also be paid to male patients with comorbidities to mitigate the onset and progression of cognitive dysfunction.

### 6.2 Lifestyle factors

Multiple meta-analyses have demonstrated that unhealthy lifestyles as risk factors for CMM (32, 33). Additionally, studies indicate that unhealthy habits such as smoking, alcohol consumption, and physical inactivity exacerbate cognitive impairment associated with older adult patients with CMM (5). CMM patients exhibit blood pressure and glucose metabolism disorders, increasing vascular endothelial fragility (34). Concurrently, physical inactivity may heighten vascular and metabolic burdens, thereby contributing to cerebral atherosclerosis and neurodegenerative changes (35). Excessive alcohol consumption and smoking cause brain damage by increasing vascular injury and mediating inflammatory responses, ultimately triggering cognitive impairment (35). Concurrently, this provides scientific evidence for the value of healthy lifestyles in mitigating CMM-related cognitive impairment. While studies have confirmed the efficacy of lifestyle interventions in improving cognitive decline in patients with single diseases, this has not yet been validated in CMM patients (36). Therefore, focusing on shared modifiable lifestyle risk factors for both CMM and cognitive decline, and adopting multidisciplinary management strategies, may represent feasible approaches to delay and improve cognitive impairment in CMM patients.

### 6.3 Depressive factors

In 2024, scholars from the Second Affiliated Hospital of Zhejiang University in China conducted a coordinated analysis of data from 14 longitudinal cohort studies across North America, South America, Europe, Africa, Asia, and Australia examining the association between CMM, depression, and cognitive function (28). They found that depression was associated with lower cognitive function scores in CMM patients in both cross-sectional and longitudinal analyses. Cognitive decline in CMM patients with comorbid depression may involve complex mechanisms related to cerebrovascular damage, neurodegeneration, and neuroinflammation (37). CMM-induced disruptions in cerebral blood flow, oxygen supply, and insulin signaling pathways accelerate brain aging. Concurrently, depression may activate pro-inflammatory mediators, leading to microvascular damage in the brain and reduced cerebral perfusion, thereby contributing to cognitive impairment. Therefore, attention should be paid not only to physiological factors but also to the impact of psychological factors on cognitive impairment in CMM patients, necessitating thorough psychological assessment and care for such individuals.

Furthermore, research on CMM suggests that polypharmacy may have a detrimental effect on cognitive function in older adults with CMM (22). Older adult patients combined with cardiovascular disease and diabetes often take multiple prescription medications simultaneously. The cumulative adverse effects of these medications may exacerbate cognitive decline in CMM patients, though this conclusion requires further basic research to substantiate. In summary, cognitive function in older adult patients with CMM is influenced by multiple factors, suggesting that age, gender, lifestyle, and depression may serve as important indicators for screening cognitive impairment or potential targets for improving cognitive function.

## 7 Public health strategies and implications

These findings bolster the public health focus on early cognitive screening and risk stratification among vulnerable populations. We propose pragmatic screening triggers, including targeted cognitive assessment for adults aged 65 years and older with two or more cardiometabolic diseases (CMDs). In addition, nurse-led integrated care models represent a highly feasible, accessible, and cost-effective approach in primary care and community settings. Nurses can undertake regular risk factor monitoring, standardized cognitive assessment, health education, and long-term follow-up, which effectively bridges gaps in current dementia prevention services and improves adherence to risk control strategies. For patients who have already experienced cognitive impairment, innovative self-management models should be developed that integrate self-health monitoring, chronic disease management, and cognitive function prevention. Patients with CMM should be encouraged to adopt proactive health strategies, supported in participating in and utilizing new technologies to aid self-management, with the goal of reducing the risk of cognitive impairment and improving quality of life. From an economic perspective, uncontrolled CMM contributes to a substantial socioeconomic burden by accelerating cognitive decline, elevating the risk of disability, and increasing both acute and long-term healthcare costs associated with cognitive impairment and dementia. Implementing targeted and cost-effective interventions—such as health education, balanced diet, regular physical activity, smoking cessation, and weight management—may effectively slow cognitive deterioration. In addition, appropriate deprescribing of potentially inappropriate medications that carry cognitive adverse effects can further prevent unnecessary cognitive decline, reduce avoidable hospitalizations and medical expenditures, and ultimately alleviate the substantial economic burden on individuals, families, and healthcare systems.

## 8 Summary and limitations

In summary, CMM directly accelerates the progression of cognitive impairment and dementia. Cognitive dysfunction in older adult patients with CMM correlates with multiple adverse outcomes and directly increases mortality. Age, gender, lifestyle, and depression are key factors influencing cognitive impairment in this population. Healthcare professionals should prioritize monitoring CMM’s impact on cognitive function in older adult patients and screen high-risk individuals based on known risk factors. Current research on CMM and cognitive function remains in its infancy. Future studies should focus on diverse population structures and disease co-occurrence patterns, emphasizing shared modifiable risk factors. Developing cross-disciplinary, multidisciplinary management teams centered on self-management strategies for comorbidities will improve cognitive impairment and advance healthy aging. This study is merely an ordinary review, not a comprehensive review. It did not systematically search all relevant literature in accordance with evidence-based methodology, thus having certain limitations.

## References

[1] Di Angelantonio E Kaptoge S Wormser D Willeit P Butterworth AS Bansal N . Association of cardiometabolic multimorbidity with mortality. JAMA. (2015) 314:52–60. doi: 10.1001/jama.2015.7008, 26151266 PMC4664176

[2] Xu X Mishra GD Dobson AJ Jones M. Progression of diabetes, heart disease, and stroke multimorbidity in middle-aged women: a 20-year cohort study. PLoS Med. (2018) 15:e1002516. doi: 10.1371/journal.pmed.1002516, 29534066 PMC5849280

[3] Zhang D Tang X Shen P Si Y Liu X Xu Z . Multimorbidity of cardiometabolic diseases: prevalence and risk for mortality from one million Chinese adults in a longitudinal cohort study. BMJ Open. (2019) 9:e024476. doi: 10.1136/bmjopen-2018-024476, 30833320 PMC6443196

[4] Liu G Wang J Zeng L Qiu Y Wang S. Relationship between cardiometabolic multimorbidity and cognitive function in Chinese elderly. Chin Nurs Res. (2023) 37:1701–6. doi: 10.12102/j.issn.1009-6493.2023.10.002

[5] Jin Y Liang J Hong C Liang R Luo Y. Cardiometabolic multimorbidity, lifestyle behaviours, and cognitive function: a multicohort study. Lancet Healthy Longev. (2023) 4:e265–73. doi: 10.1016/S2666-7568(23)00054-5, 37150183

[6] Li Q Hu H Zhang G Hu H Ou Y Huang L . Associations between cardiometabolic multimorbidity and cerebrospinal fluid biomarkers of Alzheimer’s disease pathology in cognitively intact adults: the cable study. Alzheimer's Res Ther. (2024) 16:1–11. doi: 10.1186/s13195-024-01396-w, 38321520 PMC10848421

[7] Valletta M Vetrano DL Xia X Rizzuto D Roso-Llorach A Calderon-Larranaga A . Multimorbidity patterns and 18-year transitions from normal cognition to dementia and death: a population-based study. J Intern Med. (2023) 294:326–35. doi: 10.1111/joim.13683 37306092, 37306092

[8] Dove A Marseglia A Shang Y Grande G Vetrano DL Laukka EJ . Cardiometabolic multimorbidity accelerates cognitive decline and dementia progression. Alzheimers Dement. (2023) 19:821–30. doi: 10.1002/alz.12708, 35708183

[9] Chu CS Cheng SL Bai YM Su TP Tsai SJ Chen TJ . Multimorbidity pattern and risk for mortality among patients with dementia: a nationwide cohort study using latent class analysis. Psychiatry Investig. (2023) 20:861–9. doi: 10.30773/pi.2023.0112, 37794668 PMC10555512

[10] Dove A Guo J Marseglia A Fastbom J Vetrano DL Fratiglioni L . Cardiometabolic multimorbidity and incident dementia: the Swedish twin registry. Eur Heart J. (2023) 44:573–82. doi: 10.1093/eurheartj/ehac744, 36577740 PMC9925275

[11] She R Yan ZR Wang P Liang YJ Qiu CX. Cardiometabolic and panvascular multimorbidity associated with motoric cognitive risk syndrome in older adults. J Geriatr Cardiol. (2024) 21:944–53. doi: 10.26599/1671-5411.2024.10.001, 39619359 PMC11605508

[12] Zhang H Jiang S Hao M Li Y Hu Z Jiang XY . Association of cardiometabolic multimorbidity with motoric cognitive risk syndrome in older adults. Alzheimers Dement (Amst). (2023) 15:e12491. doi: 10.1002/dad2.12491, 37937160 PMC10626031

[13] Shang X Zhu Z Zhang X Huang Y Zhang X Liu J . Association of a wide range of chronic diseases and apolipoprotein e4 genotype with subsequent risk of dementia in community-dwelling adults: a retrospective cohort study. Eclin Med. (2022) 45:101335. doi: 10.1016/j.eclinm.2022.101335, 35299656 PMC8921546

[14] Lyall DM Celis-Morales CA Anderson J Gill JM Mackay DF McIntosh AM . Associations between single and multiple cardiometabolic diseases and cognitive abilities in 474 129 UK biobank participants. Eur Heart J. (2017) 38:577–83. doi: 10.1093/eurheartj/ehw528, 28363219 PMC5381595

[15] Li X. Clinical research progress on cognitive dysfunction in stroke patients. Chin J Phys Med Rehabil. (2008) 30:784–6. doi: 10.3321/j.issn:0254-1424.2008.11.018

[16] Tian Y Shao M Wang B Ma X. Research progress on the mechanisms of cognitive dysfunction induced by cardiovascular diseases. Shandong Med J. (2021) 61:85–8. doi: 10.3969/j.issn.1002-266X.2021.05.024

[17] Yang W. Research progress on cognitive decline and dementia in elderly patients with diabetes. Chin J Diffic Complic Cases. (2017) 16:644–8. doi: 10.3969/j.issn.1671-6450.2017.06.026

[18] İmre O Caglayan C Muştu M. The relationship of cognitive dysfunction with inflammatory markers and carotid intima media thickness in schizophrenia. J Pers Med. (2023) 13:1342. doi: 10.3390/jpm13091342, 37763110 PMC10532434

[19] Liu C Liu R Tian N Fa W Liu K Wang N . Cardiometabolic multimorbidity, peripheral biomarkers, and dementia in rural older adults: the MIND-China study. Alzheimers Dement. (2024) 20:6133–45. doi: 10.1002/alz.14091, 38982798 PMC11497761

[20] Fohner AE Bartz TM Tracy RP Adams H Bis JC Djousse L . Association of serum neurofilament light chain concentration and MRI findings in older adults: the cardiovascular health study. Neurology. (2022) 98:e903–11. doi: 10.1212/WNL.0000000000013229, 34921102 PMC8901174

[21] He L de Souto BP Aggarwal G Nguyen AD Morley JE Li Y . Plasma abeta and neurofilament light chain are associated with cognitive and physical function decline in non-dementia older adults. Alzheimer's Res Ther. (2020) 12:128. doi: 10.1186/s13195-020-00697-0 33032662 PMC7545881

[22] Vassilaki M Aakre JA Cha RH Kremers WK St SJ Mielke MM . Multimorbidity and risk of mild cognitive impairment. J Am Geriatr Soc. (2015) 63:1783–90. doi: 10.1111/jgs.13612 26311270 PMC4607039

[23] Compilation Group of Chinese Expert Consensus on Safe Medication Management for Frail Elderly in Integrated Care Institutions. Chinese expert consensus on safe medication management for frail elderly in integrated care institutions (2022 edition). Chin J Health Care Med. (2022) 24:355–62. doi: 10.3969/j.issn.1674-3245.2022.05.001

[24] Zhang J Huang X Ling Y Xie X Zeng X Lyu J . Associations of cardiometabolic multimorbidity with all-cause dementia, Alzheimer’s disease, and vascular dementia: a cohort study in the UK biobank. BMC Public Health. (2025) 25:2397. doi: 10.1186/s12889-025-23352-5, 40624483 PMC12232823

[25] Debette S Seshadri S Beiser A Au R Himali JJ Palumbo C . Midlife vascular risk factor exposure accelerates structural brain aging and cognitive decline. Neurology. (2011) 77:461–8. doi: 10.1212/WNL.0b013e318227b227, 21810696 PMC3146307

[26] Writing Group on Chinese Guidelines for the Diagnosis and Treatment of Dementia and Cognitive Impairment and Cognitive Disorders Professional Committee of the Neurology Branch of the Chinese Medical Doctor Association. 2018 Chinese guidelines for the diagnosis and treatment of dementia and cognitive impairment (3): cognitive and functional assessment of dementia. Natl Med J China. (2018) 98:1125–9. doi: 10.3760/cma.j.issn.0376-2491.2018.15.002

[27] Xing X Yang X Chen J Wang J Zhang B Zhao Y . Multimorbidity, healthy lifestyle, and the risk of cognitive impairment in Chinese older adults: a longitudinal cohort study. BMC Public Health. (2024) 24:46. doi: 10.1186/s12889-023-17551-1, 38166903 PMC10762941

[28] Zhao X Xu X Yan Y Lipnicki DM Pang T Crawford JD . Independent and joint associations of cardiometabolic multimorbidity and depression on cognitive function: findings from multi-regional cohorts and generalisation from community to clinic. Lancet Reg Health West Pac. (2024) 51:101198. doi: 10.1016/j.lanwpc.2024.101198, 39308753 PMC11416683

[29] Chinese Society of Cardiology and Editorial Board of Chinese Journal of Cardiology. Chinese expert consensus on cardiovascular diseases and cognitive impairment. Chin J Cardiol. (2023) 51:455–68. doi: 10.3760/cma.j.cn112148-20230220-00096 37198116

[30] Livingston G Huntley J Sommerlad A Ames D Ballard C Banerjee S . Dementia prevention, intervention, and care: 2020 report of the lancet commission. Lancet. (2020) 396:413–46. doi: 10.1016/S0140-6736(20)30367-6, 32738937 PMC7392084

[31] Shang X Zhang X Huang Y Zhu Z Zhang X Liu J . Association of a wide range of individual chronic diseases and their multimorbidity with brain volumes in the UK biobank: a cross-sectional study. Eclin Med. (2022) 47:101413. doi: 10.1016/j.eclinm.2022.101413, 35518119 PMC9065617

[32] Jia M Peng J Liu X Liu Y Zhao H. Risk factors for cardiometabolic multimorbidity: a meta-analysis. Prev Med. (2023) 35:790–5. doi: 10.19485/j.cnki.issn2096-5087.2023.09.013

[33] Zhang J Sun Y. Prevalence and influencing factors of cardiometabolic multimorbidity in middle-aged and older adults: a meta-analysis. Chin J Geriatr Care. (2023) 21:9–15. doi: 10.3969/j.issn.1672-2671.2023.06.002

[34] Liu G Li Y Hu Y Zong G Li S Rimm EB . Influence of lifestyle on incident cardiovascular disease and mortality in patients with diabetes mellitus. J Am Coll Cardiol. (2018) 71:2867–76. doi: 10.1016/j.jacc.2018.04.027, 29929608 PMC6052788

[35] Freisling H Viallon V Lennon H Bagnardi V Ricci C Butterworth AS . Lifestyle factors and risk of multimorbidity of cancer and cardiometabolic diseases: a multinational cohort study. BMC Med. (2020) 18:5. doi: 10.1186/s12916-019-1474-7, 31918762 PMC6953215

[36] Ngandu T Lehtisalo J Solomon A Levalahti E Ahtiluoto S Antikainen R . A 2 year multidomain intervention of diet, exercise, cognitive training, and vascular risk monitoring versus control to prevent cognitive decline in at-risk elderly people (finger): a randomised controlled trial. Lancet. (2015) 385:2255–63. doi: 10.1016/S0140-6736(15)60461-5, 25771249

[37] Ryan MC Hong LE Hatch KS Gao S Chen S Haerian K . The additive impact of cardio-metabolic disorders and psychiatric illnesses on accelerated brain aging. Hum Brain Mapp. (2022) 43:1997–2010. doi: 10.1002/hbm.25769, 35112422 PMC8933252

